# TSARM-UDP: An Efficient Time Series Association Rules Mining Algorithm Based on Up-to-Date Patterns

**DOI:** 10.3390/e23030365

**Published:** 2021-03-19

**Authors:** Qiang Zhao, Qing Li, Deshui Yu, Yinghua Han

**Affiliations:** 1 School of Control Engineering, Northeastern University at Qinhuangdao, Qinhuangdao 066004, China; learner_2003@163.com; 2 College of Computer and Communication Engineering, Northeastern University at Qinhuangdao, Qinhuangdao 066004, China; 1701912@stu.neu.edu.cn (D.Y.); yhhan723@126.com (Y.H.)

**Keywords:** association rules mining, time-series, temporal relationships, up-to-date pattern, data mining

## Abstract

In many industrial domains, there is a significant interest in obtaining temporal relationships among multiple variables in time-series data, given that such relationships play an auxiliary role in decision making. However, when transactions occur frequently only for a period of time, it is difficult for a traditional time-series association rules mining algorithm (TSARM) to identify this kind of relationship. In this paper, we propose a new TSARM framework and a novel algorithm named TSARM-UDP. A TSARM mining framework is used to mine time-series association rules (TSARs) and an up-to-date pattern (UDP) is applied to discover rare patterns that only appear in a period of time. Based on the up-to-date pattern mining, the proposed TSAR-UDP method could extract temporal relationship rules with better generality. The rules can be widely used in the process industry, the stock market, etc. Experiments are then performed on the public stock data and real blast furnace data to verify the effectiveness of the proposed algorithm. We compare our algorithm with three state-of-the-art algorithms, and the experimental results show that our algorithm can provide greater efficiency and interpretability in TSARs and that it has good prospects.

## 1. Introduction

Data mining is a recently emerging technology that can mine the information behind the massive data in many domains. Through years of research in data mining, many mining methods were proposed, such as techniques for association rules, classification rules, clusters, sequential patterns, and so on. Among the numerous algorithms in data mining, association rules mining (ARM) can effectively handle quantitative data. Due to the ARM results being linguistic and easily understood and explained [1,2], it has been used in many fields, such as bio-informatics [3,4], recommender systems [5], medicine sciences [6], process industry manufacturing [7], and the economic sphere [8], to discover knowledge and play an auxiliary role in decision making [9,10].

However, in the real world, most data are always generated in a data stream form like industrial data, network data, and business data. These data often have time labels. Usually, the correlation of multiple time-series represents the nature of the data. Thus, excavating TSARs is a meaningful job. Haupt et al. [11] extracted frequent patterns from calendar schemes; then, Lucia Sacchi et al. proposed a kind of temporal association rule and the related extraction algorithm for complex patterns defined over clinical time-series. The proposed approach was based on a qualitative representation of increase, decrease, and stationery trends, which relied on the formalism of knowledge-based temporal abstractions [12]. Chen et al. [13] applied the membership function in fuzzy theory to association rules mining, and the method reflected the lifespan of an item by redefining Support and Confidence. The algorithm obtained the effective time of rules through the lifespan of each item. Furthermore, Chen et al. proposed a fuzzy time-series mining algorithm. The algorithm can effectively mine TSARs with a sliding window, but it was problematic in that the mining results related to the window size and the types of membership function were difficult to assert [14]. Two tree-based algorithms were presented in [15] to mine the frequent temporal patterns that considered not only the Support of patterns, but also their weights. Yu et al. used the information-filtering algorithm to filter the interference information and redundant information in the social network and applied fuzzy data clustering to the mining and clustering of relational data in hierarchical networks in [16]. In [17], Park et al. proposed a method for discovering the rules to describe deviant event patterns from multivariate time-series, called SAX-ARM. The algorithm first uses inverse normal transformation to convert the distribution of time-series to the normal distribution and then applies symbolic aggregate approximation to symbolize time-series for discovering frequent rules. However, most such algorithms are based on the traditional a priori algorithm [18], which discovers the frequent pattern is greater than or equal to a user-specified minimum support in a top-down level-wise process. The frequent patterns represent the expected patterns in the whole database, but may ignore some information that is usually hidden behind temporal relationship patterns.

To extract the temporal relationships in TSARs, in [19,20], the concept of a time cube and the a priori algorithm were presented to mine the temporal association rules. However, these methods do not consider multiple items between transactions, and inherent information is difficult to mine, which causes the rules to be less interpretable. A new visualization solution explicitly dealing with temporal association rules was presented in [21]. In [22], a compact FP-tree-based and divide-and-conquer algorithm was presented to mine inter-transactional association rules. Rules generated from this algorithm are interpretable, but the algorithm is susceptible to the size of the sliding window. Ruan et al. presented a framework that allowed parallel and quantitative mining of sequential patterns [23]. Kaustubh Beedkar et al. proposed a scalable, distributed sequence mining algorithm dealing with large amounts of data. The authors built a distributed framework for frequent sequence mining [24]. The description of temporal trends for the clinical domain of hemodialysis was proposed in [25], which considered specific temporal features with respect to the chosen time granularity. In [26], Hong et al. firstly proposed the concept of up-to-date patterns, which can mine rare patterns effectively. Wang et al. proposed the frequent itemset tree, and the algorithm can discover temporal association rules among multiple variables [27,28]. Lin and Hong proposed the tree structure temporal mining algorithm based on their previous up-to-date studies, and the mining result of their algorithms can discover implicit knowledge and obtain satisfactory results [29,30,31]. Recent work about graph association rule mining [32] has the potential to take temporal information into account. In [33], to resolve the issue of incremental rare association rule mining, Borah et al. presented a single-pass tree-based approach for extracting rare association rules when new data were inserted into the original database. The approach is capable of generating the complete set of frequent and rare patterns without rescanning the updated database and reconstructing the entire tree structure when new transactions are added to the existent database. However, rules mined by the above works are TSARs with a time interval, but not the whole time. Such rules are effective in a period, but not for the entire time. In some fields, such as the process industry and medical treatment, TSARs with a lifespan would be no longer effective, and TSARs with generality are more useful.

**Figure 1:**
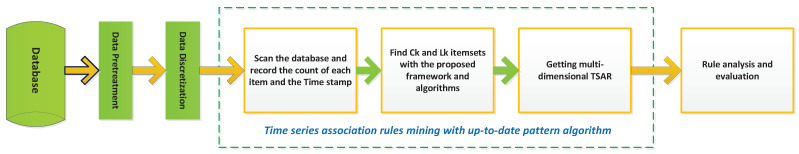
The framework of TSARM-UDP.

In this paper, to solve the problems mentioned above, a new TSAR mining framework is proposed to mine TSARs with generality. Since multiple variables need to be considered, they are divided into one-dimensional TSARM and multi-dimensional TSARM to elaborate. Furthermore, to discover implicit knowledge, we propose TSARM with up-to-date patterns (TSARM-UDP). TSARM-UDP integrates the a priori mining algorithm with the new TSAR mining framework proposed in this paper to identify TSAR from the given multivariate time-series data. Then, the UDP method is used as a reference to discover the rare frequent temporal patterns and express the mined implicit knowledge in the form of TSARs. The algorithm first scans the database to record the Count and Timelist of each item. Then, it identifies the frequent temporal patterns by the predefined *min_sup* threshold and the rare frequent temporal patterns by the UDP method. After the frequent temporal patterns have been found, TSARs can be discovered from the given time-series data. The general framework of the algorithm is shown in Figure 1.

Briefly, the novelty of this work can be highlighted as follows:

- A new TSAR mining framework is proposed in this paper to mine more rules for time-series data with higher accuracy.
- Aiming at the rare patterns that occur only for a period, the proposed algorithm can find more effective association rules.
- The proposed TSAR-UDP method can extract temporal relationships without experienced knowledge and extend the rules’ applicability to the whole dataset.

The remainder of this paper is organized as follows. The preliminaries of the proposed algorithm are given in Section 2. In Section 3, we discuss the one-dimensional TSARs and multidimensional TSARs in detail and introduce the UDP method briefly. Experimental results are given in Section 4. Conclusions and future research are given in Section 5.

## 2. Preliminaries of Our Proposed Algorithm

### 2.1. Time Series

**Definition** **1.** *Time-series* X *consists of the value of* X *in different time stamps: Timeseries(X) = (x${}_{1}$, x${}_{2}$, x${}_{3}$, …, x${}_{n}$), where* X *is a variable and x${}_{i}\left(1\leq i\leq n\right)$ is the value of* X *in the i_th time stamp.*

**Definition** **2.** *A temporal transaction consists of the values of multiple variables in the $i_{th}$ time stamp, which can be described as:*

*Temporaltransaction(i) = (V${}_{1}$, V${}_{2}$, V${}_{3}$, …, V${}_{k}$), where* $V_{i}$ *is the variable.*

### 2.2. Association Rules Mining

ARM was initially used to discover the customers’ purchasing behavior patterns through the relationships among goods in supermarkets. Today, it has become one of the most popular methods in data mining. ARM can be described briefly as follows: *D* = *Database{Trans${}_{1}$, Trans${}_{2}$, …, Trans${}_{m}$}* with *m* temporal transactions, and *I = {i${}_{1}$, i${}_{2}$, …, i${}_{k}$}* is an itemset that contains *k* variables. An association rule is an implication in the form of *X*⊂*Y*, and the general form of the rule is *Rule: X → Y*, in which *X* and *Y* are disjoint itemsets in *D*. Here, *X* is called the antecedent of the rule, and *Y* is called the consequent. There are two very important concepts in ARM called: *Support* and *Confidence*. We show the definitions of these two concepts below.

**Definition** **3.** *Support(X) describes the probability that transaction X appears in D:*

(1) $$Support\left(X\right)=P\left(X\right)=\frac{count(X)}{\left|D\right|}$$

**Definition** **4.** *Support(X → Y) describes the probability that transactions X and Y appear simultaneously in D:*

(2) Support(X→Y)=Support(X∪Y)=P(X∪Y)

**Definition** **5.** *Confidence(X → Y) describes the probability that transaction Y appears in D under the condition that X appears in D:*

(3) $$Confidence\left(X\to Y\right)=\frac{Support(X\to Y)}{Support(X)}=P\left(Y|X\right)$$

In the process of mining association rules, Lift is usually used to test the validity of rules. The rule is effective only when the Lift value of the rule is greater than one, and that rule is called a strong association rule. The formula of Lift is given below:

(4) $$Lift\left(X\to Y\right)=\frac{Support(X⋃Y)}{Support(X)*Support(Y)}=\frac{P(Y|X)}{P(Y)}$$

In this paper, we consider a rule to be strong when its *Confidence* and Lift are greater than min_sup and one, respectively.

The a priori algorithm is one of the most classic ARM algorithms; it was first proposed by Agrawal in 1993. The process of mining rules can be summarized in two parts as follows:

(1) Find all frequent items in the original log database by the predefined *min_sup*.
(2) Generate association rules in frequent items by the predefined *min_conf*.

## 3. The Proposed TSARM-UDP

In this section, a new TSARM framework is proposed to discover TSARs with generality. We divided it into one-dimensional TSARs and multidimensional TSARs to clarify the proposed framework. In the last section, a novel algorithm named TSARM-UDP is proposed to discover implicit knowledge in the form of TSARs.

### 3.1. Time Series Association Rules Mining

Understanding temporal relationships among multiple variables can help decision making, and the discovered knowledge can be inherited and learned later. However, traditional ARM mining frameworks cannot discover the temporal relationships from time-series data. To mine TSARs in time-series from a temporal database, several issues need to be solved: (1) a strict time order relationship should be considered; (2) candidate itemsets generated by the a priori method may not satisfy the downward property; (3) a new mining framework should be proposed to discover the rules with generality.

#### 3.1.1. One-Dimensional TSARs

The definition of one-dimensional TSARs proposed in this paper is given below:

**Definition** **6.** *If X occurs at time t, then Y will appear at time t + T, and the TSARs’ form can be expressed as $Rule(X\overset{T}{\to }Y)$, where T is a time constant.*

**Definition** **7.** *$TSup(X\overset{T}{\to }Y)$ describe the probability that variable X occurs at time t and variable Y occurs at time t+T,*

(5) $$TSup\left(X\overset{T}{\to }Y\right)=\frac{F(X,Y,T)}{|D|-T}$$

*where |D| is the total number of transactions in the log database.*

**Definition** **8.** *F(X,Y,T) is the total number of transaction that satisfy the following: if X appears at time t, then Y appears at time t + T.*

**Figure 2:**
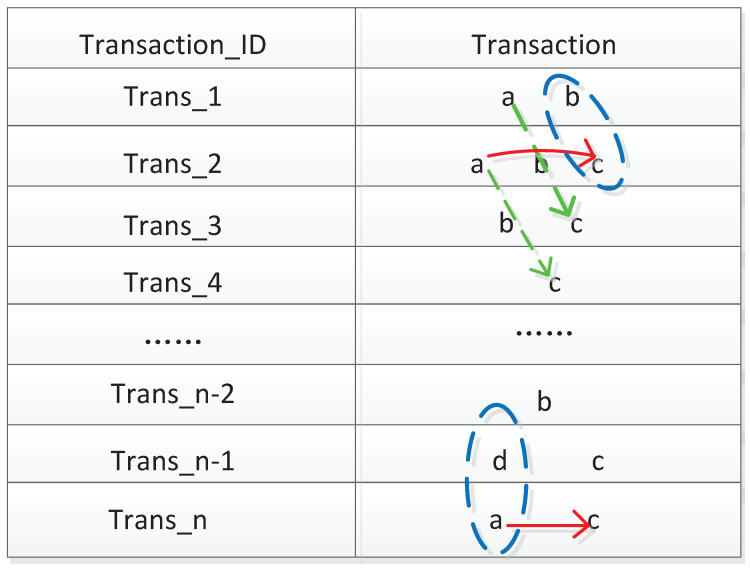
The difference of calculating Support between Formula (2) and Formula (5).

The differences in the *Support* calculation between TSARs and traditional association rules mainly lie in the calculation methods of the numerator and the denominator in Formula (5). The TSAR takes the relationship among multiple variables on a time scale into account. To further clarify the improvement, the process of calculating Support in TSAR is shown in Figure 2. We assume that there are *n* temporal transactions in the log database, and each transaction can be expressed as Trans_i. Each temporal transaction has an equal time interval.

Assuming that we are trying to determine whether the itemset (a, c) is frequent, we have to calculate the Support value of this itemset. The red solid line represents the way that the numerator in the Support formula is calculated in the traditional method, which considers that only when the item a and c occurred at the same time can be counted. The green dotted line represents the method of calculating the numerator in Formula (5), which considers the lagged time *T* (*T* is considered as two here) of item a and c for time series data in an actual industry production situation. Similarly, the total valid transactions are changed due to considering *T*, because the transactions in the blue dotted circle cannot be counted in the process of calculating TSup. Thus, the denominator in Formula (5) is |D|−T.

**Definition** **9.** *TConf in one-dimensional TSARs is defined as follows:*

(6) $$TConf\left(X\overset{T}{\to }Y\right)=\frac{TSup(X\overset{T}{\to }Y)}{Support(X)}$$

As mentioned above, to maintain the downward property in the process of mining TSARs, the candidate items’ generation method must be modified. Thus, an algorithm is presented to generate a candidate itemset in one-dimensional TSARs, which is shown in Algorithm 1. A simple example is given to illustrate the algorithm. Assuming that we have a time-series *X*, we apply the TSARM to *X* and obtain the frequent 1−itemset*L${}_{1}$*. We assume that $L_{1}=\left\{a,b,c\right\}$. Traditional association rules are used to generate candidate 2−itemsets$C_{2}=\left\{\left(a,b\right)\left(a,c\right)\left(b,c\right)\right\}$, for which (a,b) and (b,a) are regarded as the same situation. However, a clear time relationship should be noted between items when generating candidate itemsets in TSARs. Thus, the meanings of 2−itemsets(a,b) and (b,a) are different. The candidate 2−itemsets in TSARs should be $C_{2}=\left\{\left(a,b\right)\left(a,c\right)\left(b,a\right)\left(b,c\right)\left(c,a\right)\left(c,b\right)\right\}$. Note that this method is only applicable to generate candidate 2−itemsets$C_{2}$, and the method of generating $C_{k}\left(k>2\right)$ will be presented in multi-dimensional TSARs.
**Algorithm 1** Generating candidate itemsets $C_{2}$1:**Input:**2:Frequent 1_itemsets$L_{1}$3:**Output:**4:Candidate 2_itemsets$C_{2}$5:Main:6:k = 07:**for***i* in range of (0, sizeof($L_{1}$) **do**8:    **for** *j* in range of (0, sizeof($L_{1}$) **do**9:        **if** $L_{1}\left[i\right]$ == $L_{i}\left[j\right]$ **then**j++10:        **else**11:           $C_{2}\left[k\right]$ = $\left(L_{1}\left[i\right],L_{i}\left[j\right]\right)$k++12:        **end if**13:    **end for**14:**end for**15:return $C_{2}$;

#### 3.1.2. Multi-Dimensional TSARs

The multidimensional TSARs can generally be expressed as follows:

(7) $$\begin{array}{c} Rule:X_{1}\wedge X_{2}\wedge X_{3}\wedge ,\ldots ,\wedge X_{m}\\ \overset{T}{\to }Y_{1}\wedge Y_{2}\wedge Y_{3}\wedge ,\ldots ,\wedge Y_{n} \end{array}$$

Thus, if $X_{1},X_{2},X_{3},\ldots ,X_{m}$ occur at time *t*, then $Y_{1},Y_{2},Y_{3},\ldots ,Y_{n}$ will occur simultaneously at time t+T.

In terms of calculating *Support* and *Confidence*, there are some differences between multidimensional TSARs and one-dimensional TSARs. The related definitions are given below:

**Definition** **10.** *Briefly, $TSup(X_{1}\wedge X_{2}\wedge ,\ldots ,\wedge X_{m}\overset{T}{\to }Y_{1}\wedge Y_{2}\wedge ,\ldots ,\wedge Y_{n})$ describes the probability of variables $X_{1}\wedge X_{2}\wedge ,\ldots ,\wedge X_{m}$ occurring at time t simultaneously and variable $Y_{1}\wedge Y_{2}\wedge ,\ldots ,\wedge Y_{n}$ occurring at time t+T:*

(8) $$\begin{array}{c} TSup(X_{1}\wedge X_{2}\wedge \ldots ,\wedge X_{m}\overset{T}{\to }Y_{1}\wedge Y_{2}\wedge \ldots ,\wedge Y_{n})\\ =\frac{F(X_{1}\wedge X_{2}\wedge ,\ldots ,\wedge X_{m},Y_{1},Y_{2},\ldots ,Y_{n},T)}{|D|-T} \end{array}$$

**Definition** **11.** *Here, $F(X_{1}\wedge X_{2}\wedge ,\ldots ,\wedge X_{m}\overset{T}{\to }Y_{1}\wedge Y_{2}\wedge ,\ldots ,\wedge Y_{n})$ is the total number of transactions that satisfy the following: if $X_{1}\wedge X_{2}\wedge ,\ldots ,\wedge X_{m}$ appear at time t, then $Y_{1}\wedge Y_{2}\wedge ,\ldots ,\wedge Y_{n}$ appear at time t + T.*

**Definition** **12.** *TConf in multidimensional TSARs is defined as follows:*

(9) $$\begin{array}{c} TConf(X_{1}\wedge X_{2}\wedge \ldots ,\wedge X_{m}\overset{T}{\to }Y_{1}\wedge Y_{2}\wedge \ldots ,\wedge Y_{n})\\ =\frac{TSup(X_{1}\wedge X_{2}\wedge ,\ldots ,\wedge X_{m}\overset{T}{\to }Y_{1}\wedge Y_{2}\wedge ,\ldots ,\wedge Y_{n})}{Support(X_{1}\wedge X_{2}\wedge ,\ldots ,\wedge X_{m})} \end{array}$$

In many domains, the order of items is strict, especially in the process industry. We need to consider the order of items when generating candidate k−itemsets$C_{k}\left(k>2\right)$. To avoid being confused by the order of items with the increase of itemsets, we rewrite the frequent itemsets and candidate itemsets in a more specific manner. For example, if we have a frequent two-itemsets (a,c), as mentioned earlier, there is a clear order in the itemsets: item *a* is the antecedent, and item *c* is the consequent. Thus, we rewrite the frequent two-itemsets (a,c) as (a→c). The order of items can be clearly seen from this form, and this form also plays an important role in generating temporal association rules. In the remainder of the article, we use this form to represent $L_{k}$ and $C_{k}$.

Next, we explain the process of generating $C_{k+1}\left(k>2\right)$. According to the representation of frequent items $L_{k}$, we classify the itemsets in $L_{k}$ as two parts. The part on the left side of the arrow is recorded as $Class_{1}$, and the part on the right side of the arrow is recorded as $Class_{2}$. If we have the frequent itemsets $l_{1},l_{2}\in L_{k}$, it has to be ensured that $p_{1}$ equals $p_{2}$. Here, $p_{1}$ is the number of items in $Class_{1}$ of $l_{1}$, and $p_{2}$ is the number of items in $Class_{1}$ of $l_{2}$. When the antecedents (or consequents) of two itemsets are equal and satisfy the requirement that the first q−1(or p−1) items of the consequents (or antecedents) are equal, but the q−th(or p−th) item is different, any subset of candidate itemsets can be obtained by combining the two itemsets as frequent. However, if the antecedents and consequents of two itemsets are not equal, there is no guarantee that all subsets of the candidate sets are frequent. Then, we consider four situations: (1) if $p_{1}$ =1 and $Class_{2}^{1}=Class_{2}^{2}$; (2) if $q_{1}$ =1 and $Class_{1}^{1}=Class_{1}^{2}$; (3) $Class_{1}^{1}=Class_{1}^{2}$; and (4) $Class_{2}^{1}=Class_{2}^{2}$.

In the first case, the number of items in the antecedents of $l_{1}$ and $l_{2}$ is one, and the consequents of $l_{1}$ and $l_{2}$ are the same. Therefore, if $p_{1}$ and $p_{2}$ are different, the candidate itemsets $C_{k+1}$ can be obtained by combining two frequent itemsets $l_{1}$ and $l_{2}$. The second case is similar to the first case, and we will not go into detail.

In the third case, the antecedents of $l_{1}$ and $l_{2}$ are the same. Then, we have to identify whether the items in $Class_{2}$ of $l_{1}$ and $l_{2}$ satisfy the requirement that the first q−1 items are equal, but the q−th item is different. If $l_{1}$ and $l_{2}$ satisfy the requirement, then combine $l_{1}$ and $l_{2}$ to obtain $C_{k+1}$. The fourth case is similar to the third case, and we will not go into detail. For example, if we have the frequent one-itemset $L_{1}=\left\{a,b,c\right\}$ and frequent two-itemsets $L_{2}=\left\{\left(a\to c\right),\left(b\to c\right),\left(a\to c\right)\right\}$, we would like to obtain candidate itemsets $C_{3}$. Where (a→c) and (b→c) satisfy the first case, the two itemsets are combined, and we can get (a,b→c). Similarly, (a→c) and (a→b) satisfy the second case, so we can get (a→b,c). The antecedents and consequents of (a→b) and (b→c) are not equal. The subset of combining these two itemsets cannot be guaranteed to be frequent, so this case is pruned. Finally, the candidate three-itemsets are (a→b,c) and (a,b→c).

We give the pseudo code of generating candidate k−itemsets$C_{k}\left(k>2\right)$ in Algorithm 2. The rules generated in TSARs are also different from the traditional association rules. Taking the a priori algorithm as an example, unordered items are dealt with by it, which need not consider the order of items when generating rules. However, the order of items must be considered in TSARs for the reason of time-series applications. Therefore, we need to consider it when generating rules. In the step of generating rules, the order of the antecedent and consequent in frequent itemsets cannot be changed, which means the form of the rule is $Rule=Class_{1}^{i}\to Class_{2}^{i}$, and *i* means the i−th frequent itemsets in $L_{k}\left(k\geq 2\right)$.
**Algorithm 2** Generating candidate itemsets $C_{k+1}$1:**Input:**2:frequent k−itemsets$L_{k}\left(k>2\right)$3:**Output:**4:candidate itemsets $C_{k+1}$5:Main:6:Classify each item in $L_{k}$ as $Class_{1}$ and $Class_{2}$7:**for** each $l_{1}$ in $L_{k}$ **do**8:    **for** each $l_{2}$ in $L_{k}$ **do**9:        **if** $p_{1}$ == $p_{2}$ **then**10:           **if** ($p_{1}=1$) and ($Class_{2}^{1}$ = $Class_{2}^{2}$) **then**11:               **if** $p_{1}\neq p_{2}$ **then**12:                   $C_{k+1}=l_{1}⋃l_{2}$13:               **end if**14:           **end if**15:           **if** ($q_{1}=1$) and ($Class_{1}^{1}$ = $Class_{1}^{2}$) **then**16:               **if** $q_{1}\neq q_{2}$ **then**17:                   $C_{k+1}=l_{1}⋃l_{2}$18:               **end if**19:           **end if**20:           **if** $Class_{2}^{1}$ = $Class_{2}^{2}$ **then**21:               **if** ($Class_{1}^{1}\left[1\right]=Class_{1}^{2}\left[1\right])\wedge \left(Class_{1}^{1}\left[2\right]=Class_{1}^{2}\left[2\right]\right)\wedge \ldots \wedge \left(Class_{1}^{1}\left[p-1\right]=Class_{1}^{2}\left[p-1\right]\right)\wedge (Class_{1}^{1}\left[p-1\right]<Class_{1}^{2}\left[p-1\right]$) **then**22:                   $C_{k+1}=l_{1}⋃l_{2}$23:               **end if**24:           **end if**25:           **if** $Class_{1}^{1}$ = $Class_{1}^{2}$ **then**26:               **if** ($Class_{2}^{1}\left[1\right]=Class_{2}^{2}\left[1\right])\wedge \left(Class_{2}^{1}\left[2\right]=Class_{2}^{2}\left[2\right]\right)\wedge \ldots \wedge \left(Class_{2}^{1}\left[p-1\right]=Class_{2}^{2}\left[p-1\right]\right)\wedge (Class_{2}^{1}\left[p-1\right]<Class_{2}^{2}\left[p-1\right]$) **then**27:                   $C_{k+1}=l_{1}⋃l_{2}$28:               **end if**29:           **end if**30:        **else**31:           break;32:        **end if**33:    **end for**34:**end for**35:return $C_{k+1}$;

### 3.2. Up-To-Date Patterns

Mining frequent itemsets in traditional ARM exclusively relies on the *Support* threshold to determine whether an item or an itemset is frequent. However, some transactions do not occur for the whole time period. In other words, some itemsets may be frequent in a period of time, but not for the entire database. In the real world, some cases only exist in a certain period other than the whole time. For example, the abnormal conditions in the process industry only occur in a period of time. The *Support* calculation can only be used to mine frequent itemsets in the whole database, exhibiting a very small probability to mine implicit rules about abnormal conditions. Actually, we should pay more attention to TSAR with abnormal conditions, and such rules facilitate better decision-making.

In the past, Hong et al. [26] proposed the concept of up-to-date patterns (UDPs), which were frequent patterns within their up-to-date lifetime. Lin et al. also proposed an algorithm to derive up-to-date patterns from transactions [29,30,31]. One of the advantages of the UDP method is that it can mine the implicit association rules that satisfy the current *Support* threshold.

We combine Formula (10) for reference from the UDP with the a priori algorithm. The proposed mining framework is used to mine rare patterns in the form of TSARs.

(10) $$n-First\_ID+1\leq \frac{count(i)}{min\_sup}$$

Here, *n* is the total number of transactions in the log database, First_ID is the first transaction ID in Timelist(i), count(i) is the number of occurrences of item *i* in the log database, and min_sup is the minimum *Support*, which is set in advance. If item *i* satisfies (10), then put the pattern in the set of one-items from D; otherwise, decrease count(i) by one, and repeat (10) until count(i) is equal to zero or (10) is satisfied.

### 3.3. The Proposed TSARM-UDP

#### 3.3.1. Description

**Figure 3:**
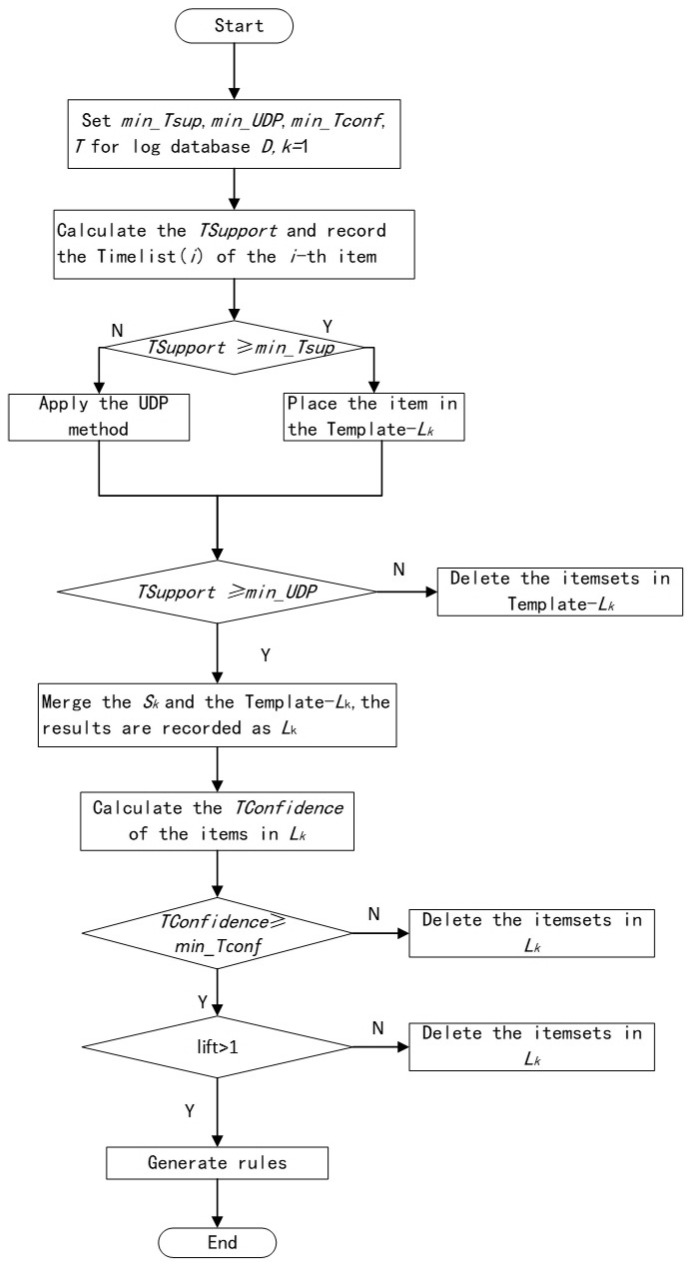
The flowchart of the proposed TSARM-UDP.

The purpose of the proposed TSARM-UDP algorithm is to mine the TSARs from time-series. The specific steps of the proposed algorithm are elaborated in the next section. The flowchart of TSARM-UDP is shown in Figure 3. Note that all data in this paper are time-series data with equal intervals.

#### 3.3.2. The Construction of the Algorithm

**Time series association rules mining with up-to-date patterns:**

Input: A log database *D* with *n* transactions stored in the order of transaction time with equal time intervals; each of them includes the transaction ID, transaction time, and items. The time *T*, the minimum support threshold min_sup, the minimum UDP threshold *min_UDP*, and the minimum confidence threshold min_conf are also included.

Output: Rules mined from the time-series.

Step 1: Scan the database *D* to generate the candidate 1−itemset$C_{1}$, and record the count value and the Timelist(i) of item *i* in the log database.

Step 2: Complete the following substeps for the items in $C_{1}$:

Substep 2.1: Calculate the *Support* of the i−th item in $C_{1}$.

Substep 2.2: If the *Support* of the item is more than min_sup, then put the item in $Template-L_{1}$. Otherwise, put the item in $S_{1}$.

Step 3: For the items *i* in $S_{1}$, complete the following substeps.

Substep 3.1: Set the First_ID(i) as the first transaction ID in the Timelist(i) of the item *i*, and verify if the item *i* satisfies Formula (10). If the item *i* satisfies Formula (10), then it will be retained in $S_{1}$ and then will be put in Template−L1.

Substep 3.2: Set the First_ID(i) as the next transaction ID in the Timelist(i) of the item *i*; decrease the count of item *i* by one; and repeat this substep until count(i) is equal to zero. If count(i) is equal to zero and the item or itemset still cannot satisfy Formula (10), then it will be deleted from $S_{1}$.

Step 4: Calculate the item or itemset as greater than or equal to min_UDP or not. If so, save the item or itemset; else, delete it.

Step 5: Combine the set $S_{1}$ and the set $Template-L_{1}$ to form $L_{1}$. Set *r* = 1, where *r* is used to keep the current number of items in the itemset to be processed.

Step 6: Generate the candidate set $C_{r+1}$ from $L_{r}$ in a similar manner to the a priori algorithm; moreover, the order of items should be considered as we mentioned above.

Step 7: Generate the frequent (r+1)-patterns $\left(L_{r+1}\right)$ from $C_{r+1}$ in a similar manner to STEPS 2 and 3.

Step 8: If the $L_{r+1}$ is null, proceed to the next step. Otherwise, jump to STEPS 5 and 6.

Step 9: Calculate the *Confidence* and *Lift* of the itemsets in the $L_{r}\left(r\geq 2\right)$ with Formulas (9) and (4). If the *Confidence* of the itemsets is greater than min_conf, then generate the rules in a manner similar to the a priori algorithm. Otherwise, delete the itemsets that cannot meet the min_conf requirement in $L_{r}$.

Step 10: Output the association rules mined from the log database.

Note that in the above algorithm, transactions in the log database must be the time-series with equal intervals.

#### 3.3.3. Set the Algorithm Parameters

Aiming at the characteristics of an actual industrial production database, we propose the TSARM-UDP algorithm. The parameters in our algorithm that need to be predefined are *TSupport*, *TConfidence*, the time *T*, the minimum *TSupport* threshold min_Tsup, the minimum UDP threshold *min_UDP*, and the minimum *TConfidence* threshold min_Tconf. Considering the differences of different datasets, these parameters should be set up according to the different situations.

#### 3.3.4. An Example

In this section, an example is given to illustrate the proposed TSARM-UDP algorithm. Table 1 shows the log database used in the example. The database contains 10 transactions and six items, denoted from *a* to *f*.

Input: *T* = 3, min_Tsup = 0.5, min_UDP = 0.1, min_Tconf = 0.4, log database *D*.

Output: Rules mined from *D*.

Step 1: Scan the database, and find the count(i) and the Timelist(i) of item *i* in *D*. Take item *a* as an example. It appears in Transactions 4, 5, and 8. Thus, count(a) is three, and Timelist(a) is {4, 5, 8}. The result of STEP 1 is shown in Table 2.

Step 2: Calculate the *TSupport* in Table 2 using Formula (8). Using item *b* as an example, the count of *b* is five. Thus, according to Formula (8), the *TSupport* of *b* is 0.5. The min_Tsup given above is 0.5, so *b* will be placed in $Template\_L_{1}$. The *TSupport* of item *c* is 0.3. This value is less than min_Tsup, so it will be placed in $S_{1}$. The *TSupport* calculation results are shown in Table 3, namely $L_{1}=\left\{b,d\right\}$ and $S_{1}=\left\{a,c,e,f\right\}$.

Step 3: For the items in $S_{1}$, the following steps are performed. Items *a* and *c* are used as examples. For item *a*, Timelist(a)={4,5,8}, so First_ID(a)=4. In addition, n=10, count(a)=3, and min_Tsup=0.5. Substitute the above parameters into Formula (10). On the left side of the inequation is 10−4+1=7. On the right side of the inequation is 3/0.5=6. The results do not satisfy the inequation, so the algorithm jumps to Substep3.2. count(a)=3−1=2, and First_ID(a)=5. Thus, the updated parameters are substituted for the inequation, and recalculate. The result still cannot satisfy the inequation. Repeat SUBSTEP 3.2. count(a)=1, and First_ID(a)=8. Then, substitute the updated parameters into the inequation, and recalculate. The result still cannot satisfy the inequation. Repeat SUBSTEP 3.2. count(a)=0. Thus, delete item *a* from $S_{1}$.

For item *c*, Timelist(c)={7,8,9}, so First_ID(c)=7, count(c)=3, n=10, and min_Tsup=0.5. The method of calculating item *a* above is used to calculate item *c*. The left side of the inequation is six, and the right side of the inequation is also six. Thus, the result satisfies the inequation, and *c* will remain in $S_{1}$.

After calculating each item in $S_{1}$, then delete the items that do not satisfy the inequation. The items that remain in $S_{1}$ are {c,e,f}.

Step 4: Calculate the count of each item in $S_{1}$, and delete the items that do not satisfy being equal to or greater than min_UDP=0.1. Update $S_{1}$.

Step 5: Combine set $S_{1}$ and set $Template\_L_{1}$ to form $L_{1}=\left\{b,c,d,e,f\right\}$. Set r=1.

Step 6: Generate the candidate set $C_{2}$ from $L_{1}$ through the method mentioned above, and the order of items should be considered. $C_{2}$ is shown in Table 4.

Step 7: Generate the frequent two-patterns $L_{2}$ in a way similar to STEPS 2 and 3. $Template\_L_{2}$ are null, and $S_{2}=\left(d\to c)(d\to e)(d\to f\right)$. Thus, $L_{2}=\left(d\to c)(d\to e)(d\to f\right)$.

Step 8: We can generate $C_{3}$ from $L_{2}$, according to the method we mentioned in the previous article. We can get $C_{3}$ = {(d→c,e)(d→c,f)(d→e,f)}, but each itemset in $C_{3}$ cannot satisfy the min_Tsup threshold and Formula (9). Thus, $L_{3}$ are null. The algorithm runs to STEP 8.

Step 9: In this step, we calculate the *TConfidence* of itemsets in $L_{2}$ by Formula (9). Taking itemsets (d→c) as an example: F(d,c,T)=3, and F(d)=7. According to Formula (9), the *TConfidence* of itemsets (d→c) is equal to 3/7. Then, we calculate the *Lift* of itemsets, Lift(d→c)=10/7, which is greater than one. Thus, Rule(d→c) is valid. The *TConfidence* and *Lift* of each itemset are given in Table 5.

As shown in Table 5, two itemsets satisfy the min_Tconf and *Lift* requirement. The rule generation method is similar to the a priori algorithm, but needs to consider the order of items and the other steps. The generated rules are given below:

$Rule\left\{1\right\}=d\overset{T}{\to }c$, with *TConfidence = 3/7*, *Lift = 10/7*

$Rule\left\{2\right\}=d\overset{T}{\to }f$, with *TConfidence = 4/7*, *Lift = 10/7*

Step 9: Output the rules.

## 4. Simulation Experiments

To better illustrate the effectiveness of the proposed TSARM-UDP algorithm, we performed several experiments on the public stock dataset [34] and real historical data of a blast furnace (BF) collected from a steel plant in China. All experiments were performed in MATLAB 2017a on a PC with a 2.5 GHz Intel Core CPU.

### 4.1. Experiment Results on the Stock Dataset

In stock market analysis, ARM is one of the widely used data mining tools to mine the underlying regularities behind the phenomenon. ARM aims to dig out the association rules (ARs) and frequent itemsets from the database. ARs reveal the relationship among different stocks, and frequent itemsets mean multiple stocks portfolio patterns in the stock dataset.

**Figure 4:**
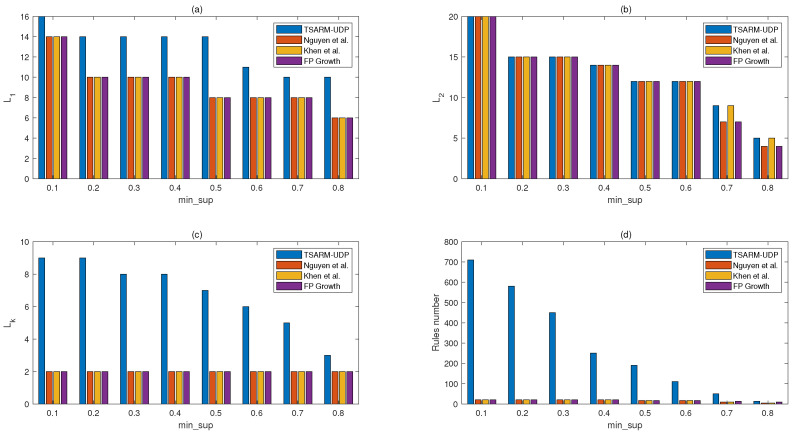
Comparisons of mining results on the stock dataset (min_conf=0.7, T=7).

**Figure 5:**
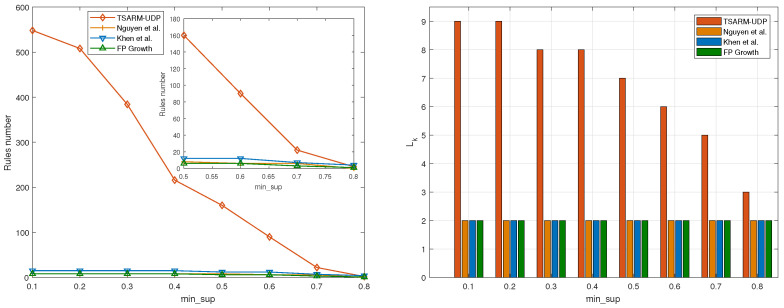
Comparisons of the rule numbers and $L_{k}$ on the stock dataset (min_conf=0.8 and T=7).

In this section, we apply the TSARM-UDP algorithm to the public stock dataset to mine time-series association rules. Furthermore, a performance comparison is done of the TSARM-UDP algorithm with other temporal algorithms presented in [35,36] and FPgrowth [22]. The mining results are shown in Figure 4 and Figure 5.

In Figure 4, we compare the mining results of the proposed algorithm and the other three algorithms on the stock dataset. min_conf is 0.7, and T=7. As shown in Figure 4, $L_{1}$, $L_{2}$, $L_{k}$, and the number of rules mined from the stock dataset by the proposed method are larger than the other state-of-the-art methods. The larger number of rules and frequent itemset means we mine more abundant information in the finite database.

In Figure 5, we give the comparison of the number of rules and $L_{k}$ produced by the four methods at different min_sup. min_conf is 0.8, and T=7. Although min_conf is improved, the algorithm proposed in this paper remains superior to other recent methods in the number of $L_{k}$ and rules.

In order to verify the accuracy rate of the rules, we divided the public stock dataset into two parts: one part was used to generate ARs, and the other one, which included the last three months of data, was used to test the forecast accuracy rate of some extra rules not obtained by traditional algorithms. The experiment was also implemented under the condition of the min conf being 0.8, min sup = 0.7, and T = 7. The test results are shown in Table 6. It can be observed that the accuracy rate of the extra rules is at a high level. The rules are as follows:

Rule{1}={(USD BASEDISE, *down*)∧(TLBASED ISE, *flat*)∧(DAX, *flat*)$\overset{7}{⟶}$(EM, *flat*)}

Rule{2}={(USD BASED ISE, *down*)∧(SP, *down*)∧(DAX, *flat*)$\overset{7}{⟶}$(EM, *flat*)}

Rule{3}={(TL BASED ISE, *flat*)∧(USD BASED ISE, *down*)∧(DAX, *flat*)∧(BOVESPA, *down*) $\overset{7}{⟶}$(EM, *flat*)}

Rule{4}={(USD BASED ISE, *down*)∧(SP, *down*)∧(DAX, *flat*)∧(NIKKEI, *down*)$\overset{7}{⟶}$(EM, *flat*)}

In Table 7, the running time of the above four methods is presented. The proposed TSARM-UDP algorithm needs a longer running time because of it mining more implicit rules. In addition, for practical application, the above running time is still within an acceptable range.

### 4.2. Experiment Results on the BF Dataset

The blast furnace (BF) plays an important role in national iron making, and the stability of the BF directly determines the quality of molten iron. The characteristics of the BF data are the time sequence, strong correlation, and rich information. Therefore, we are encouraged to use data mining methods to discover the potential relationships among multiple variables and mine implicit knowledge to assist decision-making. Moreover, since the BF is a time sensitive system, mining implicit knowledge can provide more useful information to workers for the stabilization of the furnace condition.

We applied the proposed algorithm to mine TSARs from the authentic smelting data of a BF in a steel plant in China. The data were discrete time-series with a 30-min sampling time. Based on previous research on BFs, eleven variables were chosen as the input of the algorithm. Noise was present in the BF data, so it was necessary to deal with abnormal values first. In this paper, the box diagram method was applied to remove the abnormal values in the BF data. Then, the data needed to be symbolized, and an intuitive method was used to divide the range of quantitative attributes into finite intervals of assigned symbols to form <attributes, interval>pairs. According to expert knowledge, each variable was divided into three states: descent, normal fluctuation, and ascent. The input variables and corresponding discretization intervals are shown in Table 8.

The interval division and codes are shown in Table 9, and the variable codes are shown in Table 10. To illustrate further, a simple example is presented. Assume that the blast wind volume is 3450. According to Table 8 and Table 9, three-thousand four-hundred fifty is in the range of normal fluctuation and should be encoded as 2. For the attribute of blast wind volume, the encoding number is 1 in Table 10. Therefore, the final discrete pair for this instance is 〈1,2〉. The former number represents blast wind volume, and the latter one represents normal fluctuation. According to the above method, all blast furnace data can be symbolized and used as the input for the proposed algorithm. In total, one-thousand four-hundred thirty-eight data of the authentic blast furnace were selected as the sample for time-series association rules mining. To verify the validity of this method, we compared the proposed method with that proposed in [35,36] and FP growth [22].

**Figure 6:**
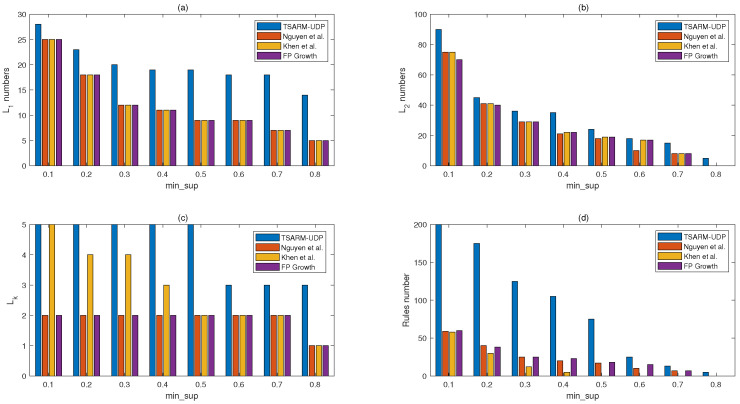
$L_{1},L_{2}$, $L_{k}$, and rule numbers comparison on the BF dataset (min_conf=0.6 and T=6).

In the first experiment, the relationships between the numbers of the frequent one-itemset for different min_sup thresholds are shown in Figure 6a. It is clear that the number of frequent itemsets discovered by TSARM-UDP is larger than the other methods.

The relationships between the numbers of the frequent two-itemsets for different min_sup thresholds are shown in Figure 6b. As shown in Figure 6b, the frequent two-itemsets without using the up-to-date method are close to zero when min_sup is 0.8, but the proposed TSARM-UDP can still mine frequent two-itemsets. The main reason is that some items may appear in some time period, but not in the whole time, and it is difficult for other methods in [35,36] and FP growth [22] to mine frequent itemsets like that. Besides, the rules with high Support are important because these situations occur frequently.

In Figure 6c, we give the maximal frequent *k*-itemsets that can be mined by the four methods. For example, when min_sup is 0.1, TSARM-UDP can mine frequent five-itemsets, but the method proposed by [35,36] and FP growth [22] can only mine two-itemsets. The method proposed by [35] can mine more itemsets than the other two methods when min_sup is less than 0.5. However, when min_sup is greater than 0.5, the maximal itemsets that can be mined by these three methods are the same. The proposed TSARM-UDP method in this paper can mine more maximal itemsets than the other three methods when min_sup takes different values. In ARM, the more frequent itemsets are mined, the more relationships between variables can be found. Therefore, the method proposed in this paper outperforms other methods in finding the relationship between multiple variables.

In Figure 6d, the number of rules produced by the four methods at different min_sup is presented, and min_conf is 0.6, T=6. As shown in this figure, compared with the other three methods, more rules were mined by the TSARM-UDP method.

**Figure 7:**
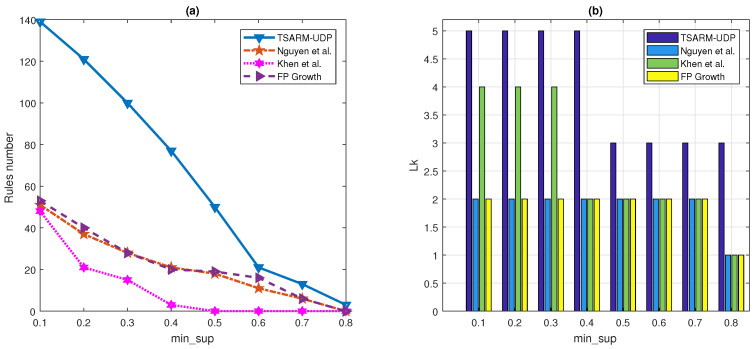
Rule numbers and $L_{k}$ comparison on the BF dataset (min_conf=0.7, T=6).

**Figure 8:**
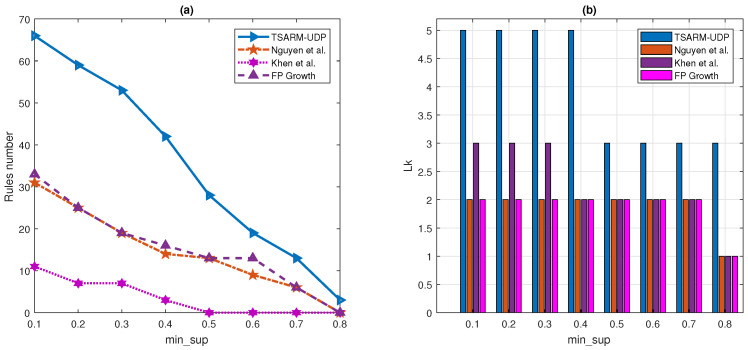
Rule numbers and $L_{k}$ comparison on the BF dataset (min_conf=0.8, T=6).

It is essential to compare the maximal itemsets $L_{k}$ and rule numbers for different min_conf values. Therefore, we compared the rule numbers and maximal itemsets $L_{k}$ that can be mined by the four methods when the min_conf values are 0.7 and 0.8, respectively. As shown in Figure 7a, more rules were mined by the proposed TSARM-UDP compared with the other methods. These rules can play a better role in decision-making. In Figure 7b, it can be clearly seen that the maximal itemsets $L_{k}$ mined by the TSARM-UDP are larger than other methods. In Figure 8, we set the min_conf=0.8 and T=6. Furthermore, the numbers of rules and $L_{k}$ that can be mined with different min_sup are compared. The experiment results shown in this figure can also draw the same conclusions as discussed in Figure 6.

Given that *T* is an artificially determined parameter, it is a novel parameter for the proposed TSARM-UDP algorithm. In the experiments we discussed above, *T* was set to six. Now, we explore the effect of different *T* values on the number of rules mined by the proposed method. The experimental results are shown in Table 11. When we chose different values for *T*, the number and content of rules were generally different. Thus, the value of *T* depends on the temporal information that users want to mine.

In Table 12, we give a comparison of the running time of each algorithm. min_conf is 0.6, and T=6. Comprehensive analyses of Figure 7 and Table 12 show that although TSARM-UDP has the longest operation time among the four methods, it can mine more effective rules. Although other method had a faster operation time than TSARM-UDP, it ignored the implicit knowledge. Moreover, in practical applications, the algorithm is generally used for offline mining, which means the requirement of operation time is not very high.

Setting min_sup=0.6,min_conf=0.6, and T=6, we used the proposed TSARM-UDP method and LTARMalgorithm [33,35] to discover knowledge from the BF data. Finally, twenty-five rules and 11 rules were obtained, respectively. The rules mined by LTARM can also be mined by TSARM-UDP. Given space constraints, we do not list all the rules mined by the TSARM-UDP method. Only a few rules that cannot be mined by LTARM are listed in Table 13, and all rules mined by the LTARM method are provided in Table 14.

### 4.3. Rules’ Evaluation

In this subsection, we compare the rules mined by the above two methods and explain some rules listed in Table 13. The analyses and explanations of the mined rules revealed that the proposed algorithm can effectively mine TSARs from BF data. Furthermore, these rules provide an effective theoretical basis for decision-making. The interval of time-series was equal to 30 min, and *T = 6*. Thus, if *X* happens, *Y* will occur after 3 h.

As shown in Table 14, the rules mined by LTARM can only assess two items, which means the method can only obtain temporal relationships between two variables in most cases. By comparing Table 13 and Table 14, the proposed TSARM-UDP method can efficiently mine the rules among multiple variables and discover the implicit rules. Moreover, more rules were mined by TSARM-UDP compared with LTARM. *Lift* shows that the implicit rules mined by TSARM-UDP are not redundant, but effective, which further proves the effectiveness of our algorithm. Next, we illustrate the meaning of some rules in Table 13.

Take Rule:{12,23,63→92,TConfidence=1,T=6} as an example. The antecedent of this rule indicates that if blast wind volume exhibits normal fluctuation, but blast wind temperature and normal blast velocity are increased, then the blast furnace bosh gas volume will fluctuate normally after 3 h.

Rule:{23,43,52,63→92,102,TConfidence=0.98817,T=6} serves as another example. If blast wind temperature, oxygen enrichment, and normal blast velocity are increased and the top temperature exhibits normal fluctuation, then the blast furnace bosh gas volume and theoretical combustion temperature will fluctuate normally after 3 h.

The temporal relationships among variables in the BF data are very complicated. Changing one variable may cause variations of other variables, so it is hard to predict how the impact on the furnace condition would happen because of the early operations. However, TSARs mined by the proposed TSARM-UDP algorithm can reveal temporal relationships among multiple items, which can provide effective evidence to help people make decisions. The rules obtained were confirmed to be valid by operators on site.

## 5. Conclusions and Further Research

In this paper, to discover the temporal relationships among multiple variables in time-series data, a new TSARM framework and a novel algorithm named TSARM-UDP are proposed. The TSARM mining framework is applied to mine TSARs and the up-to-date pattern to discover rare patterns that only appear in a period. Compared with other methods, the proposed algorithm can mine TSARs with more efficiency and better generality. Experiments on stock and BF datasets are conducted to evaluate the effectiveness and the generality of the proposed algorithms. From the results, it can be found that the proposed algorithm significantly outperforms other methods in terms of the number of frequent itemsets and the rules without depending on certain parameters. Furthermore, the mined rules have great applicability to predict stock movements with high forecast accuracy. In general, the results also show that this method could be applied in other industrial areas to make decision management more objective, reliable, and powerful.

For future work, we will consider working on future advanced versions of TSARM-UDP; e.g., updating temporal association rules according to the characteristics of a dynamic database or improving its time efficiency. We plan to work with different real-world applications and other industrial production problems.

## Figures and Tables

**Table 1:** The log database in this example.

*Transaction ID*	*Transaction Time*	*Items*
1	2018/9/1 10:00	b, d, f
2	2018/9/1 10:05	b, d, f
3	2018/9/1 10:10	d, f
4	2018/9/1 10:15	a, d
5	2018/9/1 10:20	a, b, d
6	2018/9/1 10:25	d
7	2018/9/1 10:30	c
8	2018/9/1 10:35	a, b, c
9	2018/9/1 10:40	c, f, e
10	2018/9/1 10:45	b, d

**Table 2:** The results of Timelist(i) and count(i) of each item in *D*.

*Item*	*Timelist*	*Count*
a	4, 5, 8	3
b	1, 2, 5, 8, 10	5
c	7, 8, 9	3
d	1, 2, 3, 4, 5, 6, 10	7
e	9	1
f	1, 2, 3, 9	4

**Table 3:** The results of *Timelist(i)* and *TSupport* of each item in *D*.

*Item*	*Timelist*	*TSupport*
a	4, 5, 8	0.3
b	1, 2, 5, 8, 10	0.5
c	7, 8, 9	0.3
d	1, 2, 3, 4, 5, 6, 10	0.7
e	9	0.1
f	1, 2, 3, 9	0.4

**Table 4:** The results of *Timelist(i*) and *Count(i)* of candidate 2−itemsetsT.

*Itemsets*	*Count*	*Timelist*	*Itemsets*	*Count*	*Timelist*
(b→c)	1	{5}	(d→e)	1	{6}
(b→d)	2	{1, 2}	(d→f)	1	{6}
(b→e)	0	Null	(e→b)	0	Null
(b→f)	0	Null	(e→c)	0	Null
(c→b)	0	Null	(e→d)	0	Null
(c→d)	0	Null	(e→f)	0	Null
(c→e)	0	Null	(f→b)	1	{2}
(c→f)	0	Null	(f→c)	0	Null
(d→b)	2	{2, 5}	(f→d)	3	{1, 2, 3}
(d→c)	3	{4, 5, 6}	(f→e)	0	Null

**Table 5:** The results of *TConfidence* and *Lift* in $L_{2}$.

*Itemsets*	*Confidence*	*Lift*
$d\overset{T}{\to }c$	3/7	10/7
$d\overset{T}{\to }e$	1/7	10/7
$d\overset{T}{\to }f$	4/7	10/7

**Table 6:** The accuracy rate of the rules.

*Rules*	*Accuracy Rate (%)*
Rule 1	75
Rule 2	100
Rule 3	100
Rule 4	100

**Table 7:** Comparisons of the running time of the four algorithms on the stock dataset.

*min_sup*	*TSARM-UDP*	*Nguyen et al. [35]*	*Khen et al. [36]*	*FP Tree [22]*
0.3	146.6700 s	4.1460 s	3.7000 s	2.9920 s
0.4	58.5040 s	3.4150 s	3.4640 s	2.8190 s
0.5	41.7840 s	3.2090 s	3.2700 s	2.7840 s
0.6	27.6320 s	3.1430 s	3.1900 s	2.7400 s
0.7	11.3200 s	2.8100 s	2.9940 s	2.6320 s

**Table 8:** Input variables and their corresponding discretization intervals.

*Input*	*Descent*	*Normal Fluctuation*	*Ascent*
Blast wind volume	<3400	3400∼3500	≥3500
Blast wind temperature	<1170	1170∼1190	≥1190
Blast wind pressure	<335	335∼350	≥350
Oxygen enrichment	<4400	4400∼5000	≥5000
Top temperature	<100	100∼140	≥140
Normal blast velocity	<190	190∼200	≥200
Actual blast velocity	<220	220∼230	≥230
Permeability index (PI)	<23	23∼26	≥26
Blast furnace bosh gas volume	<4400	4400∼4500	≥4500
Theoretical combustion temperature	<2200	2200∼2300	≥2300
Permeability coefficient	<6	6∼7	≥7

**Table 9:** Interval division and coding.

*Interval Division*	*Descent*	*B*	*Ascent*
Coding	1	2	3

**Table 10:** Variable coding.

*Input*	*Encoding Number*
Blast wind volume	1
Blast wind temperature	2
Blast wind pressure	3
Oxygen enrichment	4
Top temperature	5
Normal blast velocity	6
Actual blast velocity	7
Permeability index (PI)	8
Blast furnace bosh gas volume	9
Theoretical combustion temperature	10
Permeability coefficient	11

**Table 11:** Rule numbers mined by the proposed algorithm with different *T*.

*T*	*Rule Numbers*	*T*	*Rule Numbers*
(T=1)	25	(T=6)	25
(T=2)	27	(T=7)	29
(T=3)	31	(T=8)	28
(T=4)	29	(T=9)	27
(T=5)	27	(T=10)	25

**Table 12:** Comparisons of the running time of the four algorithms on the BF dataset.

*min_sup*	*TSARM-UDP*	*Nguyen et al. [35]*	*Khen et al. [36]*	*FP Tree [22]*
0.3	1.1809 × $10^{3}$ s	7.0400 s	6.8650 s	5.7770 s
0.4	438.1290 s	5.6700 s	5.8360 s	4.5320 s
0.5	162.0220 s	4.9260 s	5.1850 s	4.0640 s
0.6	45.2970 s	4.1600 s	4.6900 s	3.7080 s
0.7	29.0880 s	3.4410 s	3.4440 s	3.6820 s

**Table 13:** Example rules mined from the blast furnace data with TSARM-UDP.

*Rules*	*Confidence*	*Lift*	*T*
12,23→102	0.97952	1.1013	T=6
12,43→92	1	1.1395	T=6
23,52→92	0.98868	1.1266	T=6
32,43→102	0.90352	1.0766	T=6
12,23,52→102	0.98305	1.1053	T=6
12,23,63→92	1	1.1395	T=6
12,23→92,102	0.97952	1.1612	T=6
23,43,52→92	1	1.1395	T=6
43,52,63→92,102	0.95819	1.1359	T=6
12,23,43,52,63→102	0.98675	1.1094	T=6
23,43,52,63→92,102	0.98817	1.1715	T=6
12,23,43,63→92,102	0.97895	1.1605	T=6

**Table 14:** Rules mined from the blast furnace data with LTARM.

*Rules*	*TConfidence*	*Lift*	*T*
12→92	0.95491	1.0881	T=6
12→102	0.95049	1.0686	T=6
12→112	0.98489	1.0599	T=6
32→92	0.93668	1.0673	T=6
32→102	0.94573	1.0633	T=6
52→92	0.91852	1.0466	T=6
52→102	0.93654	1.053	T=6
52→112	0.78473	1.0429	T=6
63→92	0.95038	1.0829	T=6
63→102	0.91674	1.0307	T=6
63→112	0.80067	1.0641	T=6

## Abbreviations

The following abbreviations are used in this manuscript:

**List of abbreviations**	
*ARs*	association rules
*ARM*	association rules mining
*BF*	blast furnace
*TSARM*	time-series association rules mining algorithm
*TSARs*	time-series association rules
*UDP*	up-to-date pattern
**Notation**	
*D*	the log database
|D|	the number of transactions in the log database
*i*	an item or an itemset
*count(i)*	the number of an item’s occurrence in the database
min_sup	the minimum *Support* threshold
min_conf	the minimum *Confidence* threshold
TSup	the temporal Support
TConf	the temporal Confidence
$S_{i}$	the set used to keep the item or itemsets that cannot meet the
	min_sup requirement in the step of generating frequent itemsets
$L_{k}$	the set of frequent *i-itemsets* in the log database
$C_{k}$	the set of candidate *i-itemsets* in the log database
$Template-L_{k}$	the set used to save the item or itemsets satisfying min_sup
*Transaction_ID*	the ordinal number of the transaction in which the item is located.
*Timelist*	the set of the item’s *Transaction_ID*
First_ID	the first *Transaction_ID* in the *Timelist*
*T*	the length of time, which is predefined
$l_{i}$	a frequent itemset in $L_{k}$
$Class_{1}^{i}$	$Class_{1}$ of $l_{i}-itemset$ in $L_{k}$
$Class_{2}^{i}$	$Class_{2}$ of $l_{i}-itemset$ in $L_{k}$
$p_{i}$	the number of items in $Class_{1}$ of $l_{i}$
$q_{i}$	the number of items in $Class_{2}$ of $l_{i}$

## References

[1] Jerry C.W.L., Wensheng G., Philippe F.V., Tzung P.H., Vincent S.T. (2016). Fast algorithms for mining high-utility itemsets with various discount strategies. Adv. Eng. Inf..

[2] Galit S., Bruce P.C., Yahav I., Patel N.R., Lichtendahl K.C. (2017). Data Mining for Business Analytics: Concepts, Techniques, and Applications in R.

[3] John A., Church G.M. (2001). Aligning gene expression time-series with time warping algorithms. Bioinformatics.

[4] Mridu S., Nagwani N.K. (2018). Optimal channel selection on Electroencephalography (EEG) device data using feature re-ranking and rough set theory on eye state classification problem. J. Med. Imaging Health Inform..

[5] Zhiang W., Li C., Cao J., Ge Y. (2020). On Scalability of Association-rule-based recommendation: A unified distributed-computing framework. ACM Trans. Web..

[6] Chih-Wen C., Tsai C.F., Tsai Y.H., Wu Y.C., Chang F.R. (2020). Association rule mining for the ordered placement of traditional Chinese medicine containers: An experimental study. Medicine.

[7] Alam S., Ila M., Sickles R.C. (2000). Time series analysis of deregulatory dynamics and technical efficiency: The case of the US airline industry. Int. Econ. Rev..

[8] Fu-lai C., Fu T.C., Luk R., Ng V. (2002). Evolutionary time-series segmentation for stock data mining. Proceedings of the IEEE Congress on Evolutionary Computation, ICDM 2002.

[9] Matthews S.G., Gongora M.A., Hopgood A.A., Ahmadi S. (2013). Web usage mining with evolutionary extraction of temporal fuzzy association rules. Knowl.-Based Syst..

[10] Okolica J.S., Peterson G.L., Mills R.F., Grimaila M.R. (2018). Sequence pattern mining with variables. IEEE Trans. Knowl. Data Eng..

[11] Haupt R.L., Haupt S.E., Khosravy M., Gupta N. (2004). Practical Genetic Algorithms.

[12] Sacchi L., Larizza C., Combi C., Bellazzi R. (2007). Data mining with temporal abstractions: Learning rules from time-series. Data Min. Knowl. Discov..

[13] Chen C.-H., Lan G.C., Hong T.P., Lin S.B. (2016). Mining fuzzy temporal association rules by item lifespans. Appl. Soft. Comput..

[14] Chen C.-H., Hong T.-P., Tseng V.S. (2012). Fuzzy data mining for time-series data. Appl. Soft. Comput..

[15] Shirsath P.A., Verma V.K. (2013). A recent survey on incremental temporal association rule mining. IJITEE.

[16] Yang Y., Tang Y. (2020). The construction of hierarchical network model and wireless activation diffusion optimization model in English teaching. EURASIP J. Wirel. Commun. Netw..

[17] Park H., Jung J.-Y. (2020). SAX-ARM: Deviant event pattern discovery from multivariate time-series using symbolic aggregate approximation and association rule mining. Expert Syst. Appl..

[18] Agrawal R., Imieliński T., Swami A. Mining association rules between sets of items in large databases. Proceedings of the 1993 ACM SIGMOD International Conference on Management of Data.

[19] Ghorbani M., Abessi M. (2017). A new methodology for mining frequent itemsets on temporal data. IEEE Trans. Eng. Manag..

[20] Mantovani M., Combi C., Zeggiotti M. Discovering and analyzing trend-event patterns on clinical data. Proceedings of the 2019 IEEE International Conference on Healthcare Informatics.

[21] Combi C., Sabaini A. Extraction, analysis, and visualization of temporal association rules from interval-based clinical data. Proceedings of the 2013 Conference on Artificial Intelligence in Medicine in Europe.

[22] Qin L.X., Shi Z.Z. (2005). Research on multiple time-series inter-transaction association analysi. Comput. Eng. Appl..

[23] Ruan G., Zhang H., Plale B. Parallel and quantitative sequential pattern mining for large-scale interval-based temporal data. Proceedings of the 2014 IEEE International Conference on Big Data.

[24] Beedkar K., Gemulla R., Martens W. (2019). A unified framework for frequent sequence mining with subsequence constraints. ACM Trans. Database Syst..

[25] Combi C., Pozzi G., Rossato R. (2012). Querying temporal clinical databases on granular trends. J. Biomed. Inform..

[26] Hong T.-P., Wu Y.-Y., Wang S.-L. (2009). An effective mining approach for up-to-date patterns. Expert Syst. Appl..

[27] Wang L., Meng J., Xu P., Peng K. (2018). Mining temporal association rules with frequent itemsets tree. Appl. Soft. Comput..

[28] Wang L., Li L.L., Meng J.Y. (2018). Temporal association rules mining algorithm based on frequent item sets tree. Control Decis..

[29] Lin J.C.-W., Gan W., Hong T.P., Tseng V.S. (2015). Efficient algorithms for mining up-to-date high-utility patterns. Adv. Eng. Inform..

[30] Lin C.-W., Hong T.-P., Lu W.-H. Mining up-to-date knowledge based on tree structures. Proceedings of the 2009 International Conference of Soft Computing and Pattern Recognition.

[31] Lin C.-W., Hong T.-P. (2011). Temporal data mining with up-to-date pattern trees. Expert Syst. Appl..

[32] Namaki M.H., Wu Y., Song Q., Lin P., Ge T. (2017). Discovering graph temporal association rules. Proceedings of the 2017 ACM on Conference on Information and Knowledge Management.

[33] Borah A., Nath B. (2020). Rare association rule mining from incremental databases. Pattern Anal. Appl..

[34] ISTANBUL STOCK EXCHANGE Data Set. https://archive.ics.uci.edu/ml/d.

[35] Nguyen D., Luo W., Phung D., Venkatesh S. (2018). LTARM: A novel temporal association rule mining method to understand toxicities in a routine cancer treatment. Knowl.-Based Syst..

[36] Khan S., Parkinson S. (2018). Eliciting and utilising knowledge for security event log analysis: An association rule mining and automated planning approach. Expert Syst. Appl..

